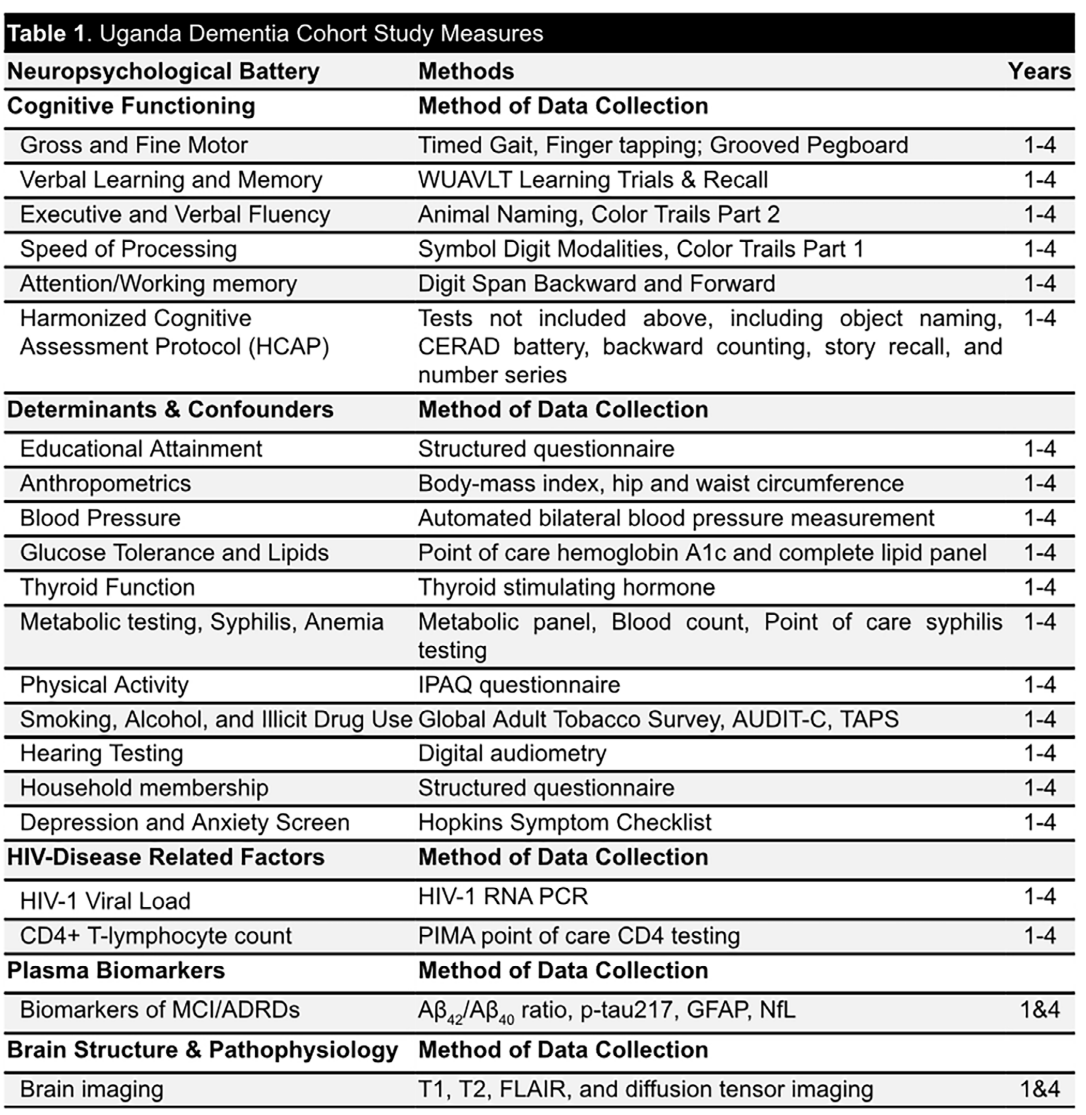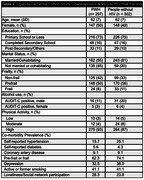# The Uganda Aging Cohort Study: Current Lessons and Future Opportunities

**DOI:** 10.1002/alz70860_100054

**Published:** 2025-12-23

**Authors:** Stephen Asiimwe, Moses Acan, Samuel Maling, Noeline Nakasujja, Flavia Atwine, Edna Tindimwebwa, Zahra Reynolds, Deanna Saylor, Alexander C Tsai, Robert Paul, Jeremy A. Tanner, Mark J Siedner

**Affiliations:** ^1^ Mbarara University of Science & Technology (MUST), Mbarara, Uganda; ^2^ Kabwohe Clinical Research Center (KCRC), Kabwohe, Uganda; ^3^ Makerere University, Kampala, Central, Uganda; ^4^ Massachusetts General Hospital (MGH), Boston, MA, USA; ^5^ Johns Hopkins Global Neurology, University Teaching Hospital, Lusaka, Lusaka, Zambia; ^6^ University of Missouri, St Louis, MO, USA; ^7^ University of Missouri, St. Louis, St. Louis, MO, USA; ^8^ Glenn Biggs Institute for Alzheimer's & Neurodegenerative Diseases, University of Texas Health Science Center, San Antonio, TX, USA

## Abstract

**Background:**

Over 25 million people with HIV (PWH) in sub‐Saharan Africa (sSA) are reaching older age in light of the success of antiretroviral therapy (ART). Older PWH are hypothesised to experience a greater burden of Alzheimer's disease and related dementias (ADRDs) due to chronic inflammatory, increased prevalence of modifiable risk factors, and increased amyloid/tau pathology. However, there are few data on the prevalence, trajectories or predictors of ADRDs in sSA, particularly among PWH. The Uganda Dementia Cohort Study (UDCS) is a prospective cohort study designed to respond to these gaps in the field.

**Method:**

The UDCS is a continuation of a 10‐year cohort study of quality of life among older adults in Uganda. PWH on suppressive ART are enrolled from the HIV clinic, while population‐based sample of age‐ and sex‐similar controls without HIV are enrolled from the same region. Each participant has had phenotyping of all 15 reported modifiable risk factors for ADRD and annual cognitive testing (Table 1)

**Result:**

This cohort has followed approximately 600 older persons with and without HIV with a mean age of 62 years. There is a higher frequency of married participants without HIV, otherwise demographics are similar by HIV serostatus (Table 2). Established risk factors for ADRDs are common in the cohort, including high prevalence rates of hypertension (20%), current or former smoking (41%), frailty (60%), and depression (40%). Rates are similar by HIV serostatus.

**Conclusion:**

The Uganda Dementia Cohort Study will enhance our understanding of ADRDs including risk/protective factors, clinical phenotypes, diagnostic tools, societal impacts, and mechanisms among older persons with and without HIV in Uganda. The cohort is expanding to include deep‐phenotyping through annual cognitive assessments, clinical examinations, plasma ADRD biomarkers, and brain imaging (Table 1). Household visits and interviews will be conducted to assess social and economic wellbeing and caretaker burden. The protocol is harmonised with other regional initiatives to facilitate future cross‐national studies. Clinical and imaging datasets and a biobank will be created to promote harmonised/cross‐national studies to better understand ADRDs in Africa. In summary, the UDCS aims to provide a rich source of data and specimens for dementia researchers in Africa and globally.